# *Leishmania (Viannia) braziliensis* isolated from the saliva of patients in a cutaneous leishmaniasis-endemic area of northeastern Brazil

**DOI:** 10.1590/0074-02760170250

**Published:** 2018-02-05

**Authors:** Maria Edileuza Felinto de Brito, Ericka Lima Almeida, Angela Cristina Rapela Medeiros, Roberto Pereira Werkhäuser, Joanna Lucia de Almeida Alexandre, Bruna Santos Lima Figueiredo Sá, Eduardo Henrique Gomes Rodrigues, Sinval Pinto Brandão-Filho

**Affiliations:** 1 Fundação Oswaldo Cruz-Fiocruz, Instituto Aggeu Magalhães, Departamento de Imunologia, Recife, PE, Brasil; 2 Universidade de Pernambuco, Recife, PE, Brasil

**Keywords:** Leishmania braziliensis, saliva, culture media

## Abstract

Several studies have described the use of non-invasive collection methods, mostly based on the detection of parasite DNA, for diagnosis. However, no *Leishmania* specimens have been isolated from saliva. Here, we report the first isolation of *Leishmania braziliensis* from the saliva of humans with cutaneous leishmaniasis but without lesions on their mucosa. The isolates were obtained from salivary fluid inoculated in hamsters and were tested by multilocus enzyme electrophoresis. Seven samples from 43 patients suspected of having the disease were identified for *in vivo* culture. These findings suggest that saliva is a clinical sample that allows the isolation of *Leishmania* sp.

American cutaneous leishmaniasis (ACL) has a wide spectrum of clinical signs and symptoms, and many species of *Leishmania* are implicated as etiologic agents of this disease (Weigle & Saraiva 1996). Clinical manifestations range from unapparent disease to skin lesions that may spontaneously resolve to multiple ulcerations and affected mucosa, which is associated with a high risk of disease recurrence and death (Costa et al. 2009).

*Leishmania braziliensis* is the primary and most prevalent pathogen associated with this disease in Brazil. This parasite is capable of producing the range of clinical manifestations, including cutaneous leishmaniasis (which may be spontaneously cured), mucosal leishmaniasis (with invasive ulcers), and disseminated cutaneous leishmaniasis (Costa et al. 2009). In a previous study, we detected 10 different kinds of zymodemes of *L. (Viannia) braziliensis*, which is the predominant species in vectors that circulate in Pernambuco, Brazil, in different clinical samples (Brito et al. 2009).

The isolation of promastigotes in culture medium allows the identification and characterisation of *Leishmania* species and therefore is of great epidemiological importance (Bensoussan et al. 2006, Rodríguez-González et al. 2006). Several studies have demonstrated non-invasive methods to collect biological samples for the diagnosis of leishmaniasis in patients suspected of having the disease (Mimore et al. 2002, Strauss-Ayali et al. 2004, Garcia et al. 2007, Figueroa et al. 2009, Boggild et al. 2011, Corvalan et al. 2011, Lombardo et al. 2012, Valencia et al. 2012). Although Corvalan et al. (2011) reported the presence of *L*. (*V*.) *braziliensis* DNA in the saliva of patients with skin lesions but without oral mucosa injury by polymerase chain reaction (PCR), they did manage to isolate the parasite from salivary fluid. Here, we report the first isolation of *L. (V.) braziliensis* from the salivary fluid of patients with ACL.

Multilocus enzyme electrophoresis (MLEE) is considered the gold standard for identifying *Leishmania* species. It allows the identification of isolates based on phenotypic polymorphisms of enzymes (Banuls et al. 2007, Schonian et al. 2010). The aims of this study were to (a) to isolate *L. braziliensis* from the saliva of patients with ACL and with no mucosal lesions, and (b) to evaluate the use of a non-invasive collection method for parasite isolation. The findings from this study suggest that saliva allows the isolation of *Leishmania* sp.

This study was performed in two municipalities: Moreno and Abreu e Lima, both of which belong to the metropolitan region of Recife, capital of Pernambuco state, Brazil. The two municipalities have no shared borders and are about 55.7 km from one another. The areas comprise 195.603 km^2^ and 125.99 km^2^ of land and have population densities of 311.94 inhabitants/km^2^ and 785.69 inhabitants/km^2^, respectively. The predominant economic activity in these regions is the cultivation of sugarcane. The houses are built close to remnants of the Atlantic Forest, with humans and domestic animals (horses, dogs, and cats) present. *Lutzomyia whitmani* is the vector primarily responsible for transmitting *L*. (*V*.) *braziliensis* in these municipalities (Brito et al. 2012).

In 2015, a total of 106 patients attending the dermatology outpatient clinic of Oswaldo Cruz Hospital, Recife, Brazil, were examined. Prior to sample collection, the participants provided informed consent using documents approved (protocol no. 038/11) by the Ethics Committee of Aggeu Magalhães Institute at the Oswaldo Cruz Foundation (AMI-PE).

Salivary fluid samples were collected in a tube, with 5-mm samples of lesions obtained from the same patients. The biological samples were transported separately in a refrigerated box. One hundred microliters of saliva and macerated tissue fragments were inoculated into hamsters’ peritonea at the Experimental Bioterium of IAM/FIOCRUZ-PE. Clinical samples (41 from tissue fragments and 43 with salivary fluid) from 106 patients were individually inoculated into 84 hamsters. After inoculation, hamsters were maintained in separate, properly identified cages with tags containing the inoculation date, name of patient, municipality of origin, and type of sample. All biological samples and syringes were also marked with the same identifiers. The animals were observed daily for ruffled or weight loss; ascites and/or death. These observations were recorded in detail.

The infected hamsters were observed for three months and then were euthanised under anaesthesia (60-100 mg/kg thiopental). Fragments of viscera (livers and spleens) of hamsters were removed and inoculated in Schneider's medium. After five days, the cultures were examined for the presence of parasites. Conventional tests (*in vivo* culture using hamsters, blood smears from hamster liver, blood smears, and PCR) were performed using other samples (biopsied tissues and swabs and scrapings from lesions). Identification of promastigotes was accomplished by MLEE, using specimens from the *Leishmania* collection of Oswaldo Cruz Foundation, Rio de Janeiro, Brazil (CLIOC) and the enzymes glucose-6-phosphate dehydrogenase (G6PDH, EC 1.1.1.49), 6-phosphogluconate dehydrogenase (6PGDH, EC 1.1.1.44), and isocitrate dehydrogenase (ICD, EC 1.1.1.42) (Cupolillo et al. 1994, Rodrigues et al. 2002). The use of animals in the study was approved by the Ethics Committee on the Use of Animals of IAM/FIOCRUZ-PE under protocol number 092/2015, according to law 2008 of 11,794 by the National Council for Animal Experimentation Control.

All patients (both male and female) who participated in the study were from areas with endemic ACL. Their ages varied, and they each presented only a single skin lesion. From seven saliva samples and 41 macerated lesion fragments obtained from 43 patients suspected of having ACL, 36 *L*. (*V*.) *braziliensis* isolates, including seven from saliva inoculated into culture medium, were obtained.

The seven patients from whom isolates were obtained from saliva were of both sexes, had an average age of 32.28 years, and each had a single skin lesion with an evolution period of approximately one to six months on the back of his/her hand, arm, or leg that measured 3.9 × 2.4 cm^2^ on average (Table). These isolates were characterised by MLEE and confirmed by PCR (Cupolillo et al. 1994, Rodrigues et al. 2002, Brito et al. 2009, Lee & Wong 2009). The results of PCR showed that the promastigotes isolated from salivary fluid, skin exudates, and tissue swabs were of the subgenus *Viannia*.

**TABLE:** Epidemiological characteristics and laboratory results obtained of saliva samples from patients with cutaneous lesions of American cutaneous leishmaniasis (ACL) endemic area in the state of Pernambuco, Northeast Brazil

Sample	Sex	Age	Lesion	Diameter (cm^2^)	Evolution (month)	Smear	PCR (Oral/Swab)	Culture	Specie
MHMO/BR/2016/JLRP2	M	13	01	hand right 5 x 2	4	+	+	+	*Leishmania (V.) braziliensis*
MHMO/BR/2016/ECP2	M	53	02	hand right 3 x 2 6 x 4	4	-	+	+	*L. (V.) braziliensis*
MHMO/BR/2016/CFO2	M	21	01	leg left 4 x 3	3	-	+	+	*L. (V.) braziliensis*
MHMO/BR/2016/SMS2	F	42	01	leg right 1,5 x 1,5	5	-	-	+	*L. (V.) braziliensis*
MHMO/BR/2016/ASP2	F	22	01	arm left 2 x 1,5	1	+	-	+	*L. (V.) braziliensis*
MHMO/BR/2016/PVS2	Μ	45	01	thigh left 2,5 x 2,5	1	+	+	+	*L. (V.) braziliensis*
MHMO/BR/2016/OMS2	F	30	01	leg right 5 x 2	1	+	+	+	*L. (V.) braziliensis*

Characterisation method used: multilocus sequence typing (MLST) (Boité et al. 2012).

From the seven patients whose saliva was positive for *L. (V.) braziliensis*, biopsy material was also obtained. All patients responded well to treatment with N-methyl glucamine at a dose of 5 to 15 mg/kg/day after only one cycle of treatment for 20 days. They were monitored for seven months, and throughout this period they did not develop additional mucosal lesions.

Classical methods to diagnose ACL have limitations in their effectiveness and applicability. Therefore, for diagnosis, it is necessary to evaluate a patient's history, clinically suspect a case, and then obtain laboratory confirmation smear, culture and molecular techniques of the presence of *Leishmania* parasites (Rodrigues et al. 2002, Brito et al. 2012). As an alternative to invasive approaches, non-invasive methods for collecting clinical samples have been developed. They are intended to be simple, practical, and more acceptable to patients. Swabbing of exudate from ulcerated lesions is a non-invasive method to collect samples from patients suspected of having ACL, and this is considered a good method to obtain samples for PCR diagnosis (Mimori et al. 2002, Figueroa et al. 2009, Boggild et al. 2011, Lombardo et al. 2012, Valencia et al. 2012). This approach allows the identification of *Leishmania* species in sub-clinical cases of disease in individuals with low parasitic burdens during treatment follow-up and to distinguish between current and past infections (Lombardo et al. 2012).

Lee & Wong (2009) showed the advantages of using saliva as a diagnostic sample for surveillance of ACL. Early detection of *Leishmania* DNA in saliva can be beneficial, both for the initiation of treatment and for research purposes. Corvalan et al. (2011) detected *L*. (*V*.) *braziliensis* DNA by PCR of saliva from patients with mucosal lesions. They concluded that this was a new, simpler option for parasitological surveys. Figueroa et al. (2009) collected swab samples of the nasal mucosa, tonsils, and conjunctiva of patients with cutaneous and mucocutaneous leishmaniasis and identified *Leishmania panamensis*, *L. braziliensis*, and *Leishmania guyannensis*. They also showed that the amount of *Leishmania* DNA in saliva decreased with treatment (Phumee et al. 2013). Saliva can also be used to detect infections with *Leishmania* species (Siriyasatien et al. 2016). However, none of these studies isolated *Leishmania* parasites from salivary fluid.

Our finding that *L. braziliensis* can be isolated from saliva might help in elucidating the escape mechanisms and/or tropism of the parasite. Further studies are needed to evaluate the performance of various methods to obtain clinical samples, including saliva, from individuals with ACL. Saliva collection in this study was effective, fast, and well accepted by patients. Furthermore, saliva has great potential for use in paediatric populations.
